# The influence of the rs1137101 genotypes of leptin receptor gene on the demographic and metabolic profile of normal Saudi females and those suffering from polycystic ovarian syndrome

**DOI:** 10.1186/s12905-018-0706-x

**Published:** 2019-01-11

**Authors:** Maha H. Daghestani, Mazin H. Daghestani, Mamoon H. Daghistani, Geir Bjørklund, Salvatore Chirumbolo, Arjumand Warsy

**Affiliations:** 1 Department of Zoology, College of Science. Director of Central Laboratory, Female Center for Scientific & Medical Colleges, King Saud University, Riyadh, Saudi Arabia; 2 Department of Obstetrics and Gynecology, Umm Al-Qura University, P.O. Box 424, Makkah, 21955 Saudi Arabia; 3 Department of Surgery, King Abdulaziz Medical City, National Guard Health Affairs, Jeddah, Kingdom of Saudi Arabia; 4 Council for Nutritional and Environmental Medicine, Mo i Rana, Norway; 5 Department of Neurological and Movement Sciences, University of Verona, Verona, Italy; 6 Central Laboratory, Female Center for Scientific and Medical Colleges, King Saud University, Riyadh, Saudi Arabia

**Keywords:** Genetic polymorphism, Leptin receptor, Polycystic ovarian syndrome

## Abstract

**Background:**

Polycystic ovarian syndrome (PCOS) is of frequent occurrence in Saudi females and is often associated with obesity, insulin resistance, hypogonadotropic hypogonadism, and infertility. Since these features are also associated with leptin receptor (LEP-R) deficiency, several studies have attempted to link *LEP-R* gene polymorphisms to PCOS.

**Methods:**

The purpose of this study is to assess the possible association of LEP-R gene polymorphism (rs1137101) with the main obesity-linked metabolic parameters in Saudi female patients affected by PCOS. A cohort of 122 Saudi female subjects, attending the outpatient’s clinics at Makkah, Saudi Arabia and diagnosed with PCOS was investigated. Metabolic parameters in serum samples, including lipidogram, glucose, leptin, ghrelin and insulin and obesity markers (BMI, W/H ratio, HOMA) were assayed and compared with values from 130 healthy female volunteers (controls). The genotyping of rs1137101 polymorphism in the leptin receptor gene by amplification (PCR) followed by DNA sequencing, was conducted in both groups (PCOS and controls).

**Results:**

Waist/hip ratio (W/H ratio), leptin serum levels and triglycerides appeared to be associated with PCOS but, aside from W/H ratio (AA s GG *p* = 0.009), this association also occurred for controls. No significant association in the leptin gene polymorphic locus rs1137101 with PCOS was seen in the results of the present study. In the control group, BMI, W/H ratio, leptin, Insulin, and HOMA-IR were significantly higher in the GG genotype compared to AA.

**Conclusion:**

Despite previous suggestion about a relationship between rs1137101, serum leptin levels, and PCOS, our studies do not show any statistical association and further investigations; possibly by also evaluating obese patients should be needed to elucidate this issue better.

## Introduction

Leptin receptor (LEP-R; OB-R; CD295), which is encoded by the *LEPR* gene, is a single-trans-membrane domain protein receptor of the cytokine receptor family [1] that binds the hormone leptin [2]. Cell membranes of a variety of cells in different organs and tissues harbor the LEP-R, signifying the role played by leptin in these tissues [3–5]. Even in the hypothalamus, the LEP-R is located in the cell membrane and is actively involved in the control of hunger and thirst and influences sleep, moods, and body temperature [6, 7]. Leptin, a polypeptide hormone, is a major player in regulating adipose tissue mass through hypothalamus effects on hunger and energy usage [6, 8]. Leptin binding to its receptor activates a series of chemical signals that influence hunger and helps in the generation of a feeling of fullness (satiety). Lean people generally have lower levels of leptin compared to their obese counterparts, and female gender, produce more leptin than their male counterpart [9]. Interest in the role of leptin in the weight control developed in the early ‘90s, and for over two decades leptin is closely linked to body weight fluctuations [10–13]. More recently it has become evident that such weight-related alterations may be a consequence of genetic variations in the *LEP-R* gene in the absence of any qualitative or quantitative leptin abnormality [14, 15]. In the *LEP-R* gene, several mutations are reported that alter the normal functional capacity of the *LEP-R* protein, resulting in an apparent “leptin receptor deficiency,” hence preventing either the binding of leptin to the receptor or the receptor from responding to the bond leptin. The consequences include excessive hunger, weight gain, and hypogonadotropic hypogonadism [16]. Severe obesity due to excessive eating (hyperphagia) begins in early childhood in LEP-R deficiency disorders and the hypogonadotropic hypogonadism, is caused by alleviated secretion of hormones involved in direct sexual development. Affected individuals experience the absence of or delay in puberty and later may suffer from infertility disorder. In a recent investigation, a statistically significant association was found between polycystic ovarian syndrome (PCOS), obesity and leptin, where the overweight and obese women with PCOS had higher serum levels of leptin compared with controls, though they have no association between PCOS and other adipokines, such as resistin and adiponectin [17]. This should prompt the researcher to deeply investigate this issue also in the influence of leptin gene polymorphism. Several polymorphisms have been reported in the *LEP-R* gene, which has been linked to a number of different disorders. One of the non-synonymous polymorphisms rs1137101 (Gln223Arg), is linked to insulin resistance, metabolic syndrome, dyslipidaemias, diabetes mellitus, obesity, besides several cancers (breast, colorectal, thyroid, prostate), preterm delivery, osteoarthritis, atherosclerosis, and ischemic heart disease [18–25]. We hypothesized that *LEP-R* gene polymorphisms might be associated with PCOS since the latter is associated with obesity, insulin resistance, hypogonadotropic hypogonadism, and infertility, all being common features of LEP-R deficiency [26]. Some associations have been reported, but the results have frequently been contradictory.

Among the Saudi women, PCOS is of frequent occurrence, though the exact frequency is yet not known (Babay Z; personal communication). In this study, the distribution of genotype and allele frequencies of the non-synonymous SNP rs1137101 (Gln233Arg) in the normal healthy Saudi females and Saudi PCOS patients, was investigated. We determined the effect of the different genotypes on the demographic and metabolic profiles. In this paper, we also present our results of association studies between rs1137101 polymorphism and PCOS and the effect of the polymorphism on demographic and metabolic profile in healthy females and those suffering from PCOS.

## Methods

### Study design and subjects

The study was conducted as a cross-sectional, case-control study and included 130 Saudi females suffering from PCOS, attending primary health clinics in Makkah, Saudi Arabia, and 122 normal healthy females also attending clinics for minor illnesses. The Institutional Review Board (IRB) at Umm Al-Qura University, Makkah, Kingdom of Saudi Arabia approved the study (IRB No. 235) and each female was required to sign an informed consent form. Patients were attending the clinics of the clinical co-investigator (MHD) and were diagnosed using the criteria set by the Rotterdam consensus [27]. The controls were age-matched (± 2 years SD), with no clinical, biochemical or hormonal abnormalities. They also had normal ultrasonic ovarian morphology and were having regular ovulatory cycles (median 25–35 days). The inclusion and exclusion criteria were as published earlier [28]. None of the individuals were on anti-diabetic or anti-obesity drugs, glucocorticoids, or other hormonal therapy. None of the females were smoking and performed daily normal physical activity.

On a specific day, each patient and control was asked to attend the clinic following overnight fasting. The weight (Kg), height (cm), waist circumference and hip circumference were measured in the standing position and used to calculate the body mass index (BMI) (Kg/cm2) and waist-hip ratio (WHR).

### Blood and serum samples

Venous peripheral blood samples were withdrawn following an overnight (12 h) fast. Fasting blood (2 ml) were collected into chilled (+ 4 °C) tubes containing 1.2 mg EDTA and aprotinin (500 KIU/ml, Trasylol, Bayer Corp., Leverkusen, Germany) for total ghrelin levels and 2 ml were drawn in fluoride tubes (gray top) for glucose estimation and 2 ml were collected in plain tubes for the biochemical analysis. All samples were immediately centrifuged, and the serum or plasma was stored at − 80 °C until further analysis. A whole blood sample collected in EDTA tubes was used to obtain genomic DNA using Gentra Systems Kit (Minneapolis, MN, cat # D5500).

### Estimation of biochemical and hormonal parameters

Blood serum was used to determine the lipids (cholesterol, triglycerides, high-density lipoprotein [HDL-C] and low-density lipoprotein [LDL-C]), applying enzymatic methods using commercial kits (Boehringer Mannheim). Estimation of leptin, ghrelin and insulin were conducted following the methods presented in Daghestani et al. [28], The homeostatic model assessment insulin resistance (HOMA-IR) and insulin secretion were calculated using the following formulas: HOMA-IR = Fasting serum insulin (μU/ml) × Fasting plasma glucose (mmol l-1)/22.5), where FI is fasting insulin and G is fasting glucose.

### Genotyping of rs1137101 polymorphism in leptin receptor gene by DNA sequencing

The DNA fragment containing the exon 6 of LEP-R gene was amplified by polymerase chain reaction (PCR) using the following primers: sense primer (5′- TATAGGCCTGAAGTGTTAGAAG-3′) and antisense primer (5′- CCCATATTTATGGGCTGAACT-3′). The conditions for PCR amplification were: initial denaturation step at 95 °C for 15 min, followed by denaturation at 95 °C for 1 min for 34 cycles, annealing at 55 °C for 60 s, and DNA extension for 1 min at 72 °C, and final extension for 10 min at 72 °C. The PCR product was checked for purity and size on agarose gel electrophoresis and was subjected to DNA sequencing, applying an ABI Big Dye Terminator protocol using ABI 3100 Avant Genetic Analyzer.

### Statistical analysis

Data for each female was entered on Excel spreadsheets and subjected to routine analysis using SPSS (Statistical Package for the Social Science, v22, Inc., Chicago, IL, USA). For each parameter mean ± SEM were obtained and results from different groups were compared using Student’s t-test or a Mann-Whitney U-test as appropriate. The patients and controls were grouped according to their genotypes, and the values of anthropometric, biochemical and hormonal parameters were calculated in each group and compared using a paired two-tailed Students ‘t’ test, separately in patients and controls. For all comparisons *p* < 0.05 was considered statistically significant.

Genotypes were manually counted in the PCOS and controls and those grouped as normal weight and obesity (PCOS and controls). The comparison of the genotype and allele frequencies in the patients and controls was obtained using the Statistical computational software available at Institute fur Humangenetik (URL: https://ihg.gsf.de/cgi-bin/hw/hwa1.pl). The odds ratio (OR), 2. 5-97.5% confidence intervals (CI), Chi-square (χ2) and *p*-value were calculated for each genotype and allele to compare the significance of the difference between the patients and controls. To assess the use of statistics in our samples and for independent samples evaluated with a t-test, the Cohen’s D test was used, calculated as the mean difference between two groups divided by the pooled standard deviation, according to:

$$ {\mathrm{Cohen}}^{\hbox{'}}\mathrm{s}\;\mathrm{d}=\left(\mathrm{M}1+\mathrm{M}2\right)/\mathrm{SDpooled} $$

Effect sizes (ES) can also be interpreted in terms of the percent of non-overlap of the treated group’s scores with those of the untreated group. In few words, the effect size gives the probability that a person selected at random from the group who were treated will have a higher score than a person picked at random from the control group. Furthermore, Hedges’s g values were computed from Cohen’s d. The parameter is an inferential measure, which is normally computed by using the square root of the mean square error (SEM) from the analysis of variance testing for differences between the two groups. To measure interaction, the ES correlation was also computed from Cohen’s d. In this case, the R-square value is the percentage of variance in the dependent variable that is accounted for by membership in the independent variable groups. For a Cohen’s d value of 1.0, the amount of variance in the dependent variable by membership in the treatment and control groups is 0,2% and *r* = 0.447 [29, 30].

## Results

The study group included 122 healthy females of Saudi origin and another group of 130 women who were diagnosed as suffering from PCOS. The demographic, metabolic and hormonal parameters in the two groups were as presented in Daghestani et al. (28). The two groups matched for their ages and BMI but showed a significant difference in the W/H ratio, where the PCOS patients had a significantly higher ratio compared to the control group. Among the lipid parameters, all parameters were elevated in the PCOS compared to the control group except HDL, which was significantly lower. Insulin, glucose, and HOMA-IR were also significantly elevated in the PCOS, but leptin, ghrelin, and HOMA-S were not different significantly when compared with the control group.

The genotype and allele frequencies of rs1137101 in the control group were compared to the results in the PCOS patients. Table 1 presents the frequencies and shows that there were no significant differences between the two groups.

**Table 1:** Comparison between the genotype and allele frequencies of rs1137101 in PCOS patients and controls

	PCOS patient	Control	OR	CI	χ^2^	*P*-value
Genotype frequency						
AA	85 (76.16)	82 (71.66)	Ref.			
AG	29 (46.69)	23 (43.68)	1.21	0.65–2.27	0.38	0.539
GG	16 (7.16)	17 (6.66)	0.90	0.43–1.91	0.06	0.799
AG + GG	45 (34.6)	40 (32.7)	1.08	0.64–1.83	0.09	0.758
Allele frequency						
A	199 (76.5)	187 (76.6)	Ref.			
G	61 (23.4)	57 (23.3)	1.00	0.66–1.51	0.00	0.976

To determine differences in the genotype and allele frequencies in patients who were normal weight and obese, the control and patients were separated into two groups based on their BMI. Table 2 presents the genotype (AA, AG, GG) and allele (A and G) frequencies of rs1137101 in the normal weight and obese PCOS patients compared to normal weight and obese controls.

**Table 2:** Comparison between the genotype and allele frequencies of rs1137101 in normal weight and obese PCOS patients and controls

Genotype/allele	Control (N)	Control (O)	OR	CI	χ^2^	*P*-value
AA	42 (37.28)	40 (31.66)	Ref.			
AG	13 (18.44)	10 (24.68)	0.76	0. 30-1.95	0.30	0.581
GG	5 (2.28)	12 (4.66)	2.40	0.77–7.44	2.39	0.122
AG + GG	18 (31.0)	22 (35.4)	1.22	0.57–2.61	0.27	0.605
A	93 (80.1)	90 (72.5)	Ref.			
G	23 (19.8)	34 (27.4)	1.52	0.83–2.79	1.91	0.167
Genotype/allele	Patient (N)	Patient (O)	OR	CI	χ^2^	*P*-value
AA	42 (38.40)	43 (37.89)	Ref.			
AG	12 (19.20)	17 (24.22)	1.38	0.59–3.24	0.56	0.454
GG	6 (2.40)	10 (4.89)	1.62	0.54–4.88	0.77	0.381
AG + GG	18 (30)	27 (38.5)	1.46	0.70–3.04	1.05	0.305
A	96 (80)	60 (42.8)	Ref.			
G	24 (20)	27 (19.2)	1.43	0.80–2.57	1.49	0.222
Genotype/allele	Control (N)	Patient (N)	OR	CI	χ^2^	*P*-value
AA	42 (39.20)	42 (38.40)	Ref.			
AG	13 (18.59)	12 (19.20)	0.92	0. 37-2.25	0.03	0.860
GG	2 (2.20)	6 (2.40)	1.20	0. 34-4.23	0.08	0.776
AG + GG	15 (25.8)	18 (29.0)	1.00	0.45–2.18	0.00	1.000
A	97 (83.6)	96 (80.0)	Ref.			
G	15 (12.9)	24 (20.0)	1.05	0.55–1.99	0.03	0.870
Genotype/allele	Control (O)	Patient (O)	OR	CI	χ^2^	*P*-value
AA	40 (32.66)	43 (37.86)	Ref.			
AG	10 (24.68)	17 (27.22)	1.58	0.64–3.85	1.02	0.311
GG	12 (4.66)	10 (4.89)	0.77	0. 30-1.99	0.28	0.596
AG + GG	22 (35.4)	27 (38.5)	1.14	0.56–2.31	0.13	0.714
A	90 (72.5)	103 (73.5)	Ref.			
G	34 (27.4)	27 (19.2)	0.95	0.55–1.64	0.03	0.856

The PCOS and control group were separated into three groups based on the genotypes: AA, AG and GG and the demographic and metabolic data were separately calculated for each genotype. The results in the different genotypes were compared, and the significance of the difference in the results was recorded. In the control group, BMI, W/H ratio, leptin, Insulin, and HOMA-IR were significantly higher in the GG genotype compared to AA. In the PCOS group statistically significant differences were observed for W/H ratio and leptin in the AA vs. GG (*p* = < 0.05), in the PCOS, while in the AA vs. AG genotypes, the triglyceride levels were higher (*p* = 0.003). However, this genotype also associated with leptin and triglyceride level in the controls (*p* = 0.01). The results are presented in Table 3.

**Table 3:** Comparison of anthropometric, hormonal and lipid parameters and glucose in PCOS and control groups carrying different genotypes of rs1137101

Parameter	Group	rs1137101 genotypes Mean ± SEM			*P*-value	
		AA	AG	GG		
BMI (kg/m2)	PCOS	30.0 ± 0.75	31.0 ± 1.37	32.5 ± 1.99	AA vs. AG	0.491
					AA vs. GG	0.162
					GG vs. AG	0.543
	Control	26.0 ± 0.72	29.4 ± 0.18	33.6 ± 2.07	AA vs. AG	0.018
					AA vs. GG	0.000
					GG vs. AG	0.181
W/H ratio	PCOS	0.84 ± 0.01	0.87 ± 0.01	0.91 ± 0.029	AA vs. AG	0.064
					AA vs. GG	0.009
					GG vs. AG	0.05
	Control	0.75 ± 0.01	0.76 ± 0.17	0.78 ± 0.016	AA vs. AG	0.040
					AA vs. GG	0.119
					GG vs. AG	0.252
Cholesterol (mmol/L)	PCOS	4.5 ± 0.11	4.35 ± 0.22	4.5 ± 023	AA vs. AG	0.530
					AA vs. GG	1.00
					GG vs. AG	0.676
	Control	3.57 ± 0.06	3.79 ± 0.11	3.72 ± 0.121	AA vs. AG	0.180
					AA vs. GG	0.313
					GG vs. AG	0.718
Triglyceride (mmol/L)	PCOS	1.05 ± 0.05	1.34 ± 0.08	1.2 ± 0.14	AA vs. AG	0.003
					AA vs. GG	0.147
					GG vs. AG	0.464
	Control	0.83 ± 0.04	0.92 ± 0.09	0.96 ± 0.09	AA vs. AG	0.001
					AA vs. GG	0.209
					GG vs. AG	0.764
HDL (mmol/L)	PCOS	1.12 ± 0.35	0.98 ± 0.03	1.07 ± 0.08	AA vs. AG	0.040
					AA vs. GG	0.613
					GG vs. AG	0.248
	Control	1.29 ± 0.03	1.30 ± 0.07	1.12 ± 0.06	AA vs. AG	0.080
					AA vs. GG	0.061
					GG vs. AG	0.248
LDL (mmol/L)	PCOS	2.55 ± 0.09	2.56 ± 0.15	2.63 ± 0.16	AA vs. AG	0.978
					AA vs. GG	0.678
					GG vs. AG	0.771
	Control	1.69 ± 0.07	1.81 ± 0.15	1.68 ± 0.13	AA vs. AG	0.101
					AA vs. GG	0.989
					GG vs. AG	0.570
Leptin	PCOS	28.2 ± 1.67	29.8 ± 3.29	38.4 ± 5.93	AA vs. AG	0.634
					AA vs. GG	0.027
					GG vs. AG	0.179
	Control	22.2 ± 1.78	29.3 ± 5.0	40.0 ± 6.48	AA vs. AG	0.060
					AA vs. GG	0.000
					GG vs. AG	0.194
Ghrelin	PCOS	0.39 ± 0.02	0.38 ± 0.16	0.32 ± 0.13	AA vs. AG	0.622
					AA vs. GG	0.053
					GG vs. AG	0.239
	Control	0.49 ± 0.01	0.39 ± 0.03	0.30 ± 0.02	AA vs. AG	0.005
					AA vs. GG	0.000
					GG vs. AG	0.042
Insulin (pmol/L)	PCOS	110.8 ± 7.7	107.4 ± 10.77	92.60 ± 14.06	AA vs. AG	0.794
					AA vs. GG	0.270
					GG vs. AG	0.412
	Control	66.7 ± 4.03	80.8 ± 7.33	95.4 ± 10.5	AA vs. AG	0.110
					AA vs. GG	0.005
					GG vs. AG	0.247
Fasting glucose	PCOS	5.09 ± 0.06	5.07 ± 0.08	5.04 ± 0.143	AA vs. AG	0.828
					AA vs. GG	0.723
					GG vs. AG	0.874
	Control	4.66 ± 0.05	4.63 ± 0.12	5.06 ± 0.12	AA vs. AG	0.522
					AA vs. GG	0.002
					GG vs. AG	0.012
HOMA-IR	PCOS	3.61 ± 1.74	3.51 ± 1.85	3.04 ± 0.489	AA vs. AG	0.788
					AA vs. GG	0.274
					GG vs. AG	0.448
	Control	2.04 ± 0.13	2.44 ± 0.24	3.16 ± 0.37	AA vs. AG	0.139
					AA vs. GG	0.002
					GG vs. AG	0.110

Subject’s distribution and statistical power were assessed by further tests, by evaluating the effect size on our sampling procedures. Table 4 describes the outputting results. Most of the comparisons (78.8%) showed a small non-overlapping percentage, calculated on Cohen’s d-effect size and demonstrate that for certain parameters (e.g., triglycerides SNP rs1137101 AA vs. AG) Cohen’s d and its co-related Gates’ delta and Hedges g, showed the same population overlapping between PCOS subjects and controls (see Table 4, 29.59 and 30.26%, respectively), assessing the sampling process performed in the study. Yet, this correspondence has not been observed for leptin (SNP rs1137101 AA vs. GG, see Table 4), what might also explain our negative data for leptin polymorphism association with PCOS, inducing out further research to improve cases and controls recruitment.

**Table 4:** Subjects distribution and statistical power assessed by Cohen’s d, Gates’ delta and Hedges’ g for evaluating the effect size on our sampling procedures. Describes the outputting Results

Parameter	Group	SNP rs 1137101 Mean ± S.D.			Cohen’s *d* effect size		Gates’ delta	Hedges’ *g*	interaction r	
BMI		**AA**	**AG**	**GG**	***d***	***% of non overlap***				N.
	PCOS	30.0 ± 8.55	31.0 ± 15.62	32.5 ± 22.69	0.484528 0.1437 0.048298	31.68% small 11.06% small 3.72% small	0.503145 0.80402 0.048119	0.483966 0.141525 0.048304	0.235 0.072 0.024	AA AG GG
	control	26.0 ± 7.95	29.4 ± 1.99	33.6 ± 22.86						
W/H ratio	PCOS	0.84 ± 0.114	0.87 ± 0.114	0.91 ± 0.33	0.803443 0.082545 0.491572	47.60% large 6.35% small 32.44% small	0.818182 0.058511 0.738636	0.802985 0.083939 0.487248	0.372 0.041 0.239	AA AG GG
	control	0.75 ± 0.110	0.76 ± 1.88	0.78 ± 0.176						
Cholesterol	PCOS	4.50 ± 0.114	4.35 ± 2.51	4.50 ± 2.62	1.963678 0.315219 0.374844	82.06% large 22.38% small 25.68% small	1.409091 5.090909 0.58209	1.993963 0.310312 0.371382	0.712 0.155 0.184	AA AG GG
	control	3.57 ± 0.66	3.79 ± 0.110	3.72 ± 1.34						
Triglycerides	PCOS	1.05 ± 0.57	1.34 ± 0.91	1.20 ± 1.60	0.432079 0.441714 0.180392	29.59% small 30.26% small 13.26% small	0.5 0.424242 0.242424	0.430339 0.442309 0.179118	0.212 0.216 0.090	AA AG GG
	control	0.83 ± 0.44	0.92 ± 0.99	0.96 ± 0.99						
Chol-HDL	PCOS	1.12 ± 3.86	0.98 ± 0.34	1.07 ± 0.91	0.062058 0.537644 0.062902	4.78% small 35.48% medium 4.84% small	0.515152 0.415584 0.075758	0.061102 0.543535 0.062592	0.031 0.261 0.031	AA AG GG
	control	1.29 ± 0.33	1.30 ± 0.77	1.12 ± 0.66						
Chol-LDL	PCOS	2.55 ± 1.03	2.56 ± 1.71	2.63 ± 1.82	0.94574 0.446357 0.580451	54.18% large 30.57% small 36.96% medium	1.116883 0.454545 0.664336	0.941487 0.446103 0.578266	0.460 0.219 0.278	AA AG GG
	control	1.69 ± 0.77	1.81 ± 1.65	1.68 ± 1.43						
Leptin	PCOS	28.2 ± 19.0	29.8 ± 37.52	38.4 ± 67.61	0.310027 0.01059 0.022983	22.01% small 0.84% small 1.77% small	0.304569 0.009053 0.022356	0.310206 0.015693 0.023003	0.153 0.0053 0.011	AA AG GG
	control	22.2 ± 19.7	29.3 ± 55.23	40.0 ± 71.57						
Ghrelin	PCOS	0.39 ± 0.22	0.38 ± 1.82	0.32 ± 1.48	0.57496 0.007646 0.018903	36.69% medium 5.89% small 1.46% small	0.909091 0.30303 0.090909	0.569518 0.007534 0.01862	0.275 0.0038 0.009	AA AG GG
	control	0.49 ± 0.110	0.39 ± 0.33	0.30 ± 0.22						
Insulin	PCOS	110.8 ± 87.8	107.4 ± 122.8	92.6 ± 160.3	0.633596 0.255755 0.020043	40.49% medium 18.80% small 1.54% small	0.991011 0.328557 0.024247	0.627686 0.254158 0.019942	0.303 0.128 0.01	AA AG GG
	control	66.7 ± 44.5	80.8 ± 80.96	95.4 ± 115.98						
Fasting glucose	PCOS	5.09 ± 0.68	5.07 ± 0.91	5.04 ± 1.63	0.695314 0.388114 0.013485	42.71% medium 26.59% small 1.04% small	0.781818 0.333333 0.015152	0.693 0.380341 0.01344	0.328 0.190 0.007	AA AG GG
	control	4.66 ± 0.55	4.63 ± 1.32	5.06 ± 1.32						
HOMA-R	PCOS	3.61 ± 19.8	3.51 ± 21.09	3.04 ± 5.57	0.111846 0.07119 0.024558	8.61% small 5.48% small 1.89% small	1.097902 0.403774 0.02934	0.110116 0.070112 0.024441	0.056 0.036 0.012	AA AG GG
	control	2.04 ± 1.43	2.44 ± 2.65	3.16 ± 4.09						

## Discussion

The *LEP-R* gene has been extensively investigated in several populations, and the association has been investigated with a number of diseases including obesity, preterm delivery, recurrent spontaneous abortion and different cancers [16–25]. Several polymorphisms have been investigated, and rs1137101 has been commonly associated with several disorders. Our results showed that *LEP-R* gene SNP rs1137101 do not show any association with obesity either in the healthy normal Saudi population or the females suffering from PCOS. The only important association was reported for the waist/hip ratio (W/H ratio), while the association with lipidogram and leptin was also observed for non-PCOS individuals. Reports showing possible leptin-W/H ratio associations were suggested either for healthy people or several disorders and aging [31–34].

However, despite some recent conclusions on obese patients [35, 36] and cancer [35, 37], no report was yet published about the possible association of the SNP rs1137101 at the leptin gene. Recent reports showed that obesity should play a major role in the pathogenesis of PCOS through the insulin, leptin and endocannabinoid receptor, and by an association between insulin receptor polymorphism and PCOS was observed [38]. Furthermore, an association of the leptin gene SNPs (Gln223Arg and Pro1019Pro) with PCOS was recently reported in a Korean population [39]. Despite this interesting evidence, our data seem to confirm previous reports showing no association between leptin gene single nucleotide polymorphism and PCOS, though PCOS is frequently associated with insulin resistance and obesity and these conditions are linked to leptin resistance and expression in the leptin receptor. Past analyses demonstrated that patients diagnosed with PCOS did not show any mutation of the leptin coding exons, while the amino acid variants in exon 2, 4 and 12, through a single-stranded conformational polymorphism (SSCP) analysis, followed by sequencing, did not show any association between leptin and PCOS [40], suggesting that much more insightful evidence should be gained by further studies. The results of this study highlight an important point, i.e. the polymorphic change resulting from the substitution of an A by G, changing the codon, CAG to CGG, and replacing Gln, a neutral amino acid by Arg, a basic amino acid, brings about an effect thereby affecting the value of different anthropometric and biochemical parameters, both in normal control and PCOS patients. The exact mechanism by which these changes are brought about needs functional and in-silico studies, whereby the mechanism of leptin-LER-R interaction can be evaluated.

Finally, since our study did not show any statistical association between rs1137101 and PCOS, further investigation, possibly by also evaluating obese patients should be needed to elucidate this issue better.

## Conclusion

In conclusion, the results of this study do not show any association between rs1137101 in the LEP-R gene and PCOS development in Saudi females. Association was observed with W/H ratio, leptin level and the lipidogram in the PCOS females. However, the association with leptin and lipidogram was also observed in the non-PCOS females. Further studies are warranted to determine the mechanism of interaction between leptin and its receptor, which results when Gln, a neutral amino acid, is replaced by Arg, a basic amino acid. Furthermore, whether there is any association between other SNPs in *LEP-R* and PCOS development in Saudis requires further detailed studies.

## Abbreviations

BMI (Kg/cm2): Body Mass Index
CI: 96% confidence intervals
ECLlA: Electrochemiluminescence immunoassay
EDTA: Ethylene diamine tetraacetate
EIA: Enzyme immunoassay
G: Fasting glucose
HDL: High-density lipoprotein
HOMA-IR: Homeostatic model assessment insulin resistance
LDL: Low-density lipoprotein
*LEPR* gene: Leptin receptor gene
LEP-R, OB-R, CD295: Leptin receptor
OR: Odds Ratio
PCOS: Polycystic ovarian syndrome
PCR: Polymerase chain reaction
SEM: Standard Error of the Mean
SNP: Single Nucleotide Polymorphism
SSCP: Single-stranded conformational polymorphism
W/H: Waist/hip ratio
χ2: Chi-square
